# Influenza A/H1N1 2009 Pandemic and Respiratory Virus Infections, Beijing, 2009–2010

**DOI:** 10.1371/journal.pone.0045807

**Published:** 2012-09-20

**Authors:** Yaowu Yang, Zhong Wang, Lili Ren, Wei Wang, Guy Vernet, Gláucia Paranhos-Baccalà, Qi Jin, Jianwei Wang

**Affiliations:** 1 MOH Key Laboratory for Systems Biology of Pathogens and Christophe Mérieux Laboratory, IPB, CAMS-Fondation Mérieux, Institute of Pathogen Biology (IPB), Chinese Academy of Medical Sciences (CAMS) & Peking Union Medical College (PUMC), Beijing, China; 2 Fondation Mérieux, Lyon, France; 3 Peking Union Medical College Hospital (PUMCH), Beijing, China; 4 China National Accreditation Service for Conformity Assessment (CNAS), Beijing, China; University of Hong Kong, Hong Kong

## Abstract

To determine the role of the pandemic influenza A/H1N1 2009 (A/H1N1 2009pdm) in acute respiratory tract infections (ARTIs) and its impact on the epidemic of seasonal influenza viruses and other common respiratory viruses, nasal and throat swabs taken from 7,776 patients with suspected viral ARTIs from 2006 through 2010 in Beijing, China were screened by real-time PCR for influenza virus typing and subtyping and by multiplex or single PCR tests for other common respiratory viruses. We observed a distinctive dual peak pattern of influenza epidemic during the A/H1N1 2009pdm in Beijing, China, which was formed by the A/H1N1 2009pdm, and a subsequent influenza B epidemic in year 2009/2010. Our analysis also shows a small peak formed by a seasonal H3N2 epidemic prior to the A/H1N1 2009pdm peak. Parallel detection of multiple respiratory viruses shows that the epidemic of common respiratory viruses, except human rhinovirus, was delayed during the pandemic of the A/H1N1 2009pdm. The H1N1 2009pdm mainly caused upper respiratory tract infections in the sampled patients; patients infected with H1N1 2009pdm had a higher percentage of cough than those infected with seasonal influenza or other respiratory viruses. Our findings indicate that A/H1N1 2009pdm and other respiratory viruses except human rhinovirus could interfere with each other during their transmission between human beings. Understanding the mechanisms and effects of such interference is needed for effective control of future influenza epidemics.

## Introduction

Since its discovery in Mexico and the United States in April 2009, the pandemic influenza A/H1N1 2009 (A/H1N1 2009pdm) has spread globally [1], [2]. As of August 1st, 2010, more than 214 countries and territories had reported laboratory-confirmed cases, and over 18,449 deaths had been attributed to A/H1N1 2009pdm [3]. The pandemic caused by A/H1N1 2009pdm also altered the epidemic patterns of seasonal influenza viruses [4], [5]. Based on the surveillance data from Europe and America, influenza virus epidemics peaked in winter/spring, 2009−2010, 14−17 weeks earlier than in previous years [6]–[11].

Although in 2010, the World Health Organization (WHO) declared the A/H1N1 2009pdm-mediated influenza pandemic to be over, A/H1N1 2009pdm continues to circulate widely and has replaced seasonal H1N1 as the seasonal virus [12]. Studies by groups from different countries or territories have reported various patterns and dynamics of the pandemic caused by A/H1N1 2009pdm [5], [13]–[17]; however, the epidemic features of A/H1N1pdm, particularly its epidemic pattern in the context of epidemics of many other respiratory viruses, have not been addressed fully.

Epidemiological studies have shown that epidemics of influenza viruses may affect the epidemics of other common respiratory viruses. For example, in France, A/H1N1pdm postponed the timing of the seasonal 2009−2010 respiratory syncytial virus (RSV) epidemic [16], [18], whereas the human rhinovirus (HRV) epidemic may have delayed the pandemic of A/H1N1pdm [17]. These observations indicate competition or interference between influenza A viruses and other respiratory viruses.

As a newly identified virus, it is uncertain what factors have affected the epidemic of A/H1N1 2009pdm and what influence the A/H1N1 2009pdm has on the epidemic of other common respiratory viruses responsible for acute respiratory tract infections (ARTIs). Understanding of the epidemic patterns and dynamics of respiratory viruses is critical. Such information could enable policy-makers to predict the trends of future epidemic and to prevent potential outbreaks [19], [20].

To characterize the A/H1N1 2009pdm epidemic pattern and to assess its impact on seasonal influenza viruses and other common respiratory viruses, we here characterized the epidemic pattern of influenza virus and other common respiratory virus infections during the first year of the A/H1N1 2009pdm epidemic in Beijing by comparing the data from the prior three years by using samples collected from adult patients with ARTIs. We also present the clinical and demographic features of A/H1N1 2009pdm infection in Beijing by using seasonal influenza and non-influenza ARTIs as controls. Our data enhances our understanding of the epidemiological features of A/H1N1 2009pdm and can aid in monitoring and controlling future epidemics.

## Materials and Methods

### Clinical Samples

Nasal and throat swab specimens were collected from patients with ATRIs at the Fever Outpatient Clinic Department at the Peking Union Medical College Hospital (PUMCH), Beijing, China. According to the regulations for ARTI management in China, all patients with acute fever must be screened at this department before they are assigned to a specific medical department in a hospital. The criteria of the patients enrolled in this study were as follows to include suspected viral ARTIs: ≥14 years of age, acute fever (≥38°C), respiratory symptoms such as cough or wheezing, and normal or low leukocyte count [21]. One nasal swab and one throat swab were taken from each patient upon the first visit and were put into a vial of viral transport medium (VTM; Copan, Brescia, Italy). Rrespiratory specimens were obtained from a total of 7,776 patients with ARTIs from May 2006 through May 2010, 259 (3.3%) patients had lower ARTIs (LRTIs, e.g. pneumonia) and 7,517 (96.7%) had upper ARTIs (URTIs, e.g. rhinitis, pharyngitis, or laryngitis). Patients’ ages ranged from 14 to 97 years (mean: 35.1 years, median: 30.0 years). The study included 3,559 male (45.8%) and 4,217 female (54.2%) patients (Table 1). The specimens were stored at −80°C prior to use. Part of these specimens had been used for etiological and epidemiological investigations on viral ARTIs within different time interval (some data unpublished) [21]–[26]. All specimens used in this study were obtained with written informed consent from all participants or guardians on behalf of the minors/children participants and were coded prior to analysis to ensure anonymity. This study was approved by the Medical Ethic Review Board of the Institute of Pathogen Biology, Chinese Academy of Medical Sciences (Beijing, China).

**Table 1:** Demographic characteristics of sampled patients.

Parameters	Year				
	2006−2007	2007−2008	2008−2009	2009−2010	2006−2010
No. of patients	2945	1838	1372	1621	7776
Age range (year)	14−97	14−97	14−87	14−90	14−97
No. of age 14−25	973 (33.0) a	572 (31.1)	484 (35.3)	559 (34.5)	2588 (33.3)
No. of age 26−65	1753 (59.5)	1119 (60.9)	823 (60.0)	966 (59.6)	4661 (59.9)
No. of age >65	212 (7.2)	143 (7.8)	64 (4.7)	93 (5.7)	512 (6.6)
No. of age unknown	7 (0.2)	4 (0.2)	1 (0.1)	3 (0.2)	15 (0.2)
Age mean/median (year)	35.6/30.0	35.9/30.0	34.1/29.0	33.9/29.0	35.1/30.0
Gender (M/F)	1335/1610 (45.3/54.7)	836/1002 (45.5/54.5)	631/741 (46.0/54.0)	757/864 (46.7/53.3)	3559/4217 (45.8/54.2)
URTIs/LRTIs	2806/139 (95.3/4.7)	1780/58 (96.8/3.2)	1316/56 (95.9/4.1)	1615/6 (99.6/0.4) b	7517/259 (96.7/3.3)
Respiratory viruses-positive	1159 (39.4)	571 (31.1)	433 (31.6)	700 (43.2)	2863 (36.8)

a Numbers in parentheses are the percentages of infection in total samples.

b ÷2 = 36.801−64.224, *P*<0.05.

For viral nucleic acid detection, total nucleic acids (DNA and RNA) were extracted from each clinical specimen using the NucliSENS® easyMAG™ apparatus (bioMérieux, Marcy L’Etoile, France) according to the manufacturer’s protocol.

### Clinical and Demographic Data Collection

For each enrolled patient, detailed demographic data and clinical information (including clinical presentations, clinical diagnosis, and laboratory tests) were collected by the clinicians when the clinical samples were taken, using a standard form. All the data collected were entered into an electronic database for statistical analysis.

### Typing and Subtyping of Influenza Viruses

Influenza A, B, and C viruses were detected by a multiplex RT-PCR as previously described [21]. The specimens positive for influenza A virus were further subtyped into three hemagglutinin (HA) and three neuraminidase (NA) subtypes (A/H1N1 2009pdm, seasonal H1N1, and H3N2) using a four-tube multiplex Taqman probe-based real-time PCR as described previously [27].

### Detection of Multiple Respiratory Viruses

In addition to influenza viruses, respiratory viruses were screened in clinical samples as described elsewhere [21]. Briefly, multiplex nested RT-PCR assays were used to detect human parainfluenza virus type 1−4 (HPIV 1−4), enterovirus (EV), human rhinovirus (HRV), and respiratory syncytial virus (RSV), while one-step RT-PCR or PCR assays were used to detect human coronaviruses (HCoVs; including 229E, OC43, NL63 and HKU1), human metapneumovirus (HMPV), and adenovirus (AdV).

### Statistical Analysis

Demographic and laboratory parameters, clinical features, and detection rates of respiratory viruses between patient groups were compared using McNemar’s chi-square test (÷2-test) for categorical variables and Student’s *t*-test for peripheral blood tests. *P*<0.05 was considered significant.

## Results

### Demographic Characterization of the Sampled Population

To exclude the bias caused by population sampling, we first analyzed the basic demographic characteristics, such as age, gender, and clinical features, of the enrolled patients. We found no significant changes in the average age, median age, or gender ratio between the 2009/2010 study year and previous study years (Table 1), with mean ages of 33.9−35.9 years, median ages of 29.0−30.0 years, and 45.3%−46.7% males in each year (÷2 = 0.149−0.789, *P>*0.05). In addition, the percentages of the three age groups (14−25, 26−65, and >65 years) did not change obviously among these study years (÷2 = 0.002−0.1.720, *P>*0.05). These data indicate that the influence of basic demographic characteristics, including patient age and gender, on the results obtained in this study can be excluded.

Furthermore, most of patients sampled had upper respiratory tract infections (URTIs) in each study year (from 95.3% to 99.6%) (Table 1). Notably, the rates of LRTIs were 3.2%−4.7% from 2006 through 2009 in contrast to 0.4% in the 2009/2010 year (÷2 = 36.801−64.224, *P*<0.05).

### Overall Detection of Respiratory Viruses and Subtyping of Influenza Viruses

In our analysis, 2,863 (36.8%) samples tested positive for at least one type of respiratory virus examined. Two or more types of viruses were detected in 129 (1.7%) samples. A total of 1,854 (23.8%) specimens were positive for influenza virus. Among those, 1,430 (77.1%) were positive for influenza A virus, 405 (21.8%) for influenza B virus, and 19 (1.0%) for influenza C virus. The influenza A virus positive specimens were further subtyped into 569 (38.8%) H3N2, 525 (36.8%) seasonal H1N1, and 261 (18.2%) A/H1N1 2009pdm. The remaining 75 (5.2%) specimens that tested positive for influenza A virus could not be subtyped by our approach. In addition, 1,145 (14.7%) specimens were positive for other common respiratory viruses, including HRV (487, 42.5%), HPIV (215, 18.8%), EV (186, 16.2%), HCoV (87, 7.6%), RSV (68, 5.9%), AdV (65, 5.7%), and HMPV (37, 3.2%) (Table 2).

**Table 2:** Detection of respiratory viruses in patients with ARTIs.

Parameters	Age group (years)				Total
	14−25	26−65	>65	Unknown	
Patients tested (no.)	2588	4661	512	15	7776
IFV A (H3N2)	172 (6.7) a	353 (7.6)	43 (8.4)	0 (0.0)	569 (7.3)
IFV A (H1N1)	186 (7.2)	317 (6.8)	21 (4.1)	2 (13.3)	525 (6.8)
IFV A (H1N1) 2009	127 (4.9)	132 (2.8)	0 (0.0)	2 (13.3)	261 (3.4)
IFV B	119 (4.6)	265 (5.7)	20 (3.9)	1 (6.7)	405 (5.2)
IFV C	9 (0.3)	10 (0.2)	0 (0.0)	0 (0.0)	19 (0.2)
Not be subtyped of IFV A	20 (0.8)	50 (1.1)	5 (1.0)	0 (0.0)	75 (1.0)
HRV	196 (7.6)	262 (5.6)	28 (5.5)	1 (6.7)	487 (6.3)
HPIV	70 (2.7)	124 (2.7)	21 (4.1)	0 (0.0)	215 (2.8)
EV	79 (3.1)	98 (2.1)	8 (1.6)	1 (6.7)	186 (2.4)
HCoV	25 (1.0)	38 (0.8)	23 (4.5)	1 (6.7)	87 (1.1)
RSV	10 (0.4)	46 (1.0)	12 (2.3)	0 (0.0)	68 (0.9)
AdV	38 (1.5)	26 (0.6)	1 (0.2)	0 (0.0)	65 (0.8)
HMPV	9 (0.3)	25 (0.5)	3 (0.6)	0 (0.0)	37 (0.5)
Total of viruses detection	1060 (35.3)	1746 (58.2)	185 (6.2)	8 (0.3)	2999

a Numbers in parentheses are the percentages of infection in total samples.

### Distinctive Pattern of Influenza Epidemic during 2009/2010

**Figure 1:**
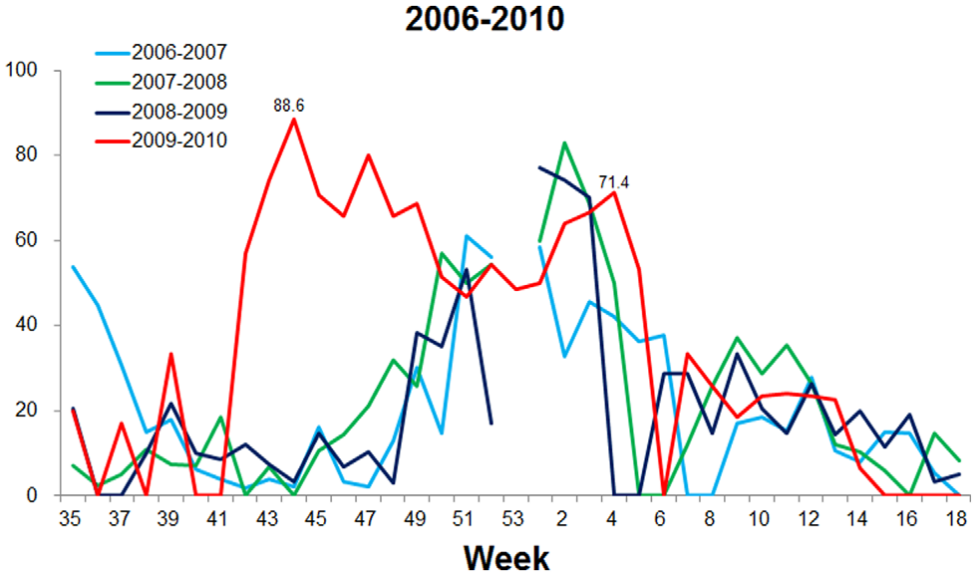
Comparison of the peaks of of influenza viruses in the epidemic seasons from 2006 to 2010. The detection rates (% positive) of influenza viruses in clinical samples tested per week are shown. Because winter/spring is the usual epidemic season of influenza in Beijing, data obtained between week 35 of one year and week 18 of the following year are presented.

**Figure 2:**
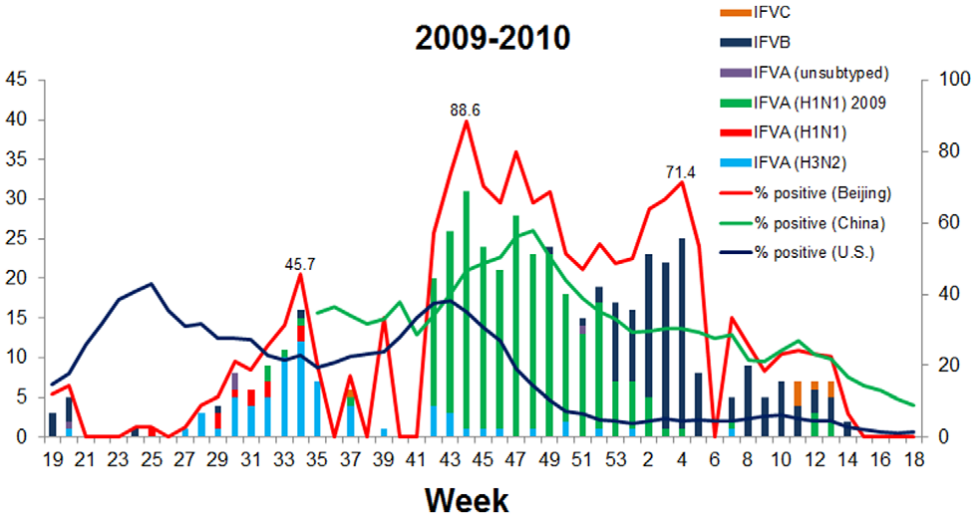
Genotyping and subtyping of influenza viruses, 2009−2010. The influenza virus-positive samples collected during week 19 of 2009 through week 18 of 2010 were typed and subtyped using a multiplex RT-PCR and a multiplex Taqman probe-based real-time PCR as described in *Materials and Methods*. Surveillance data from the CNIC and from the US CDC [6], [7] are shown for reference.

Based on our surveillance data during 2006−2009, the seasonal epidemic peak of influenza viruses usually occurred at week 51 to week 2 of the following year in Beijing (Figure 1). However, due to the pandemic of A/H1N1 2009pdm, the influenza peak appeared at week 44 (88.6%) in the winter/spring 2009−2010 in Beijing, 7−10 weeks earlier than in the winter/spring seasons during 2006−2009 (Figure 1). The peak in Beijing occurred also four weeks earlier than the national data reported by Chinese National Influenza Center (CNIC) (Figure 2).

**Figure 3:**
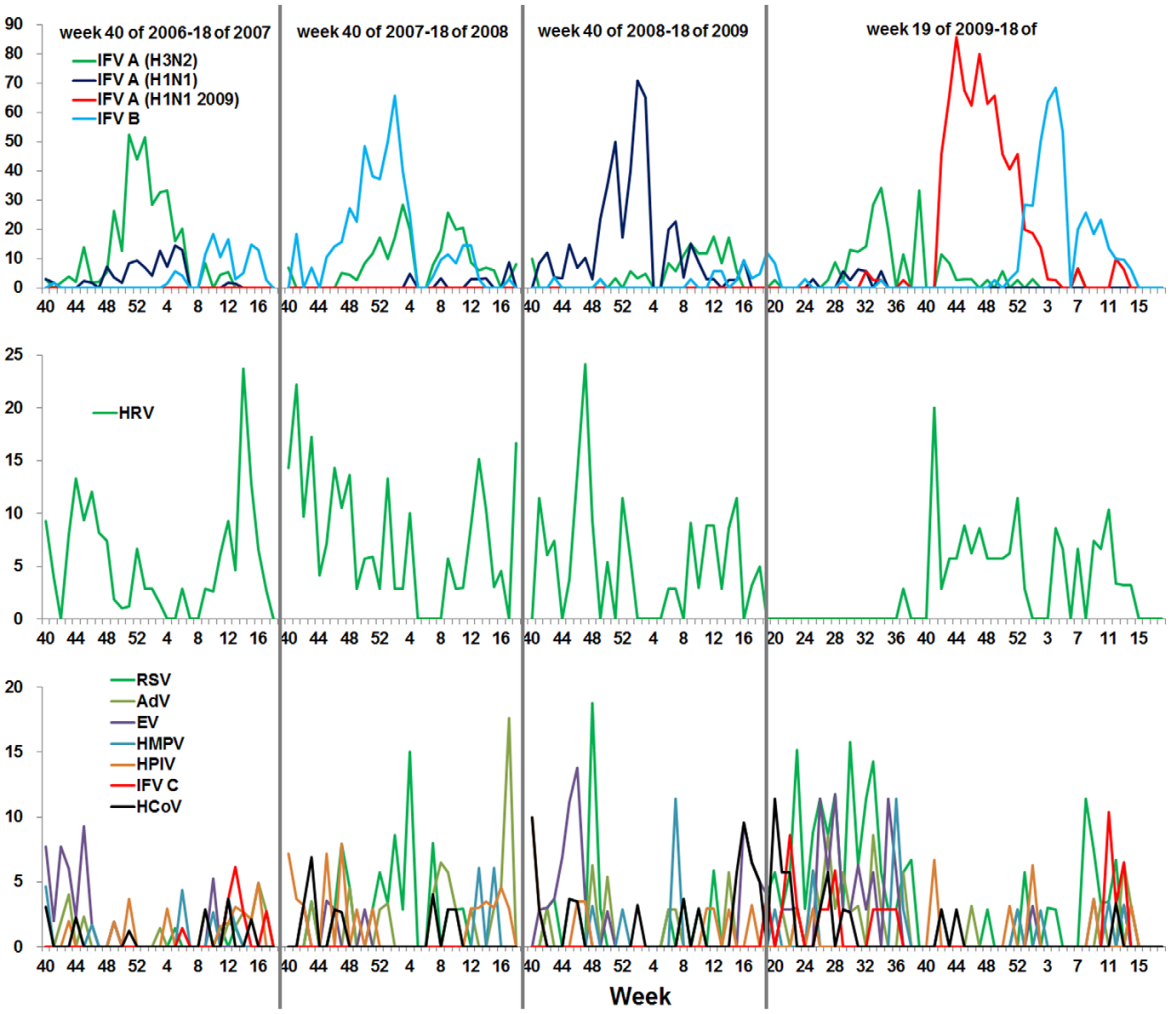
Positive rates of multiple respiratoty viruses in the winter/spring of 2006−2009 and whole year of 2009−2010. Respiratory viruses in each sample collected from week 40 of one year to week 18 of the next year in 2006−2009 and week 19 of 2009 to week 18 of 2010 were detected using multiplex nested RT-PCR, one-step RT-PCR, and PCR assays as described in *Materials and Methods*. Weekly curves of positive rate of common respiratory viruses, including A/H1N1 2009pdm, H3N2, H1N1, influenza B, C, HRV, HPIV, EV, HCoV RSV, AdV, and HMPV viruses in winter/spring 2006−2009 and annual 2009−2010 are shown.

To evaluate the effects of the A/H1N1 2009pdm on the epidemic of seasonal influenza viruses, the prevalence of influenza viruses were investigated by genotyping and subtyping samples collected from week 19 (May 4−10) of 2009, when the A/H1N1 2009pdm virus was first detected in China, through week 18 (May 3−9) of 2010 in Beijing. Notably, a smaller peak (45.7%) caused by seasonal H3N2 was observed at week 34 after the initial detection of a A/H1N1 2009pdm case in China, during weeks 27 through 37 of the summer of 2009 (Figure 2 and 3). Surprisingly, subsequent to the A/H1N1 2009pdm peak (85.7%, week 44), an influenza B virus peak (68.6%) was observed at week 4 of 2010 (Figure 2 and 3), overlapping with the pandemic peak caused by A/H1N1 2009pdm (Figure 1, 2, and 3). In contrast, during the winter/spring 2006−2009, epidemic curves of influenza viruses showed single peaks caused by one major influenza type or subtype (H3N2 in 2006/2007, influenza B virus in 2007/2008, and H1N1 in 2008/2009) (Figure 3).

### Prevalence of Multiple Respiratory Virus Infections in 2009/2010 Influenza Season

To determine if the main pandemic phase of A/H1N1 2009pdm overlapped with respiratory viral infections during week 40 of 2009 through week 18 of 2010 (winter/spring in 2009−2010), detection rates were compared with those in the previous epidemic seasons (winter/spring in 2006−2009) (Figure 1, 2 and 3). The results suggest that, in contrast to the observations made in previous influenza seasons, A/H1N1 2009pdm did not obviously interfere with the epidemic of HRV, which was influenced by seasonal influenza viruses (such as H3N2, H1N1, and influenza B virus) in the winter/spring 2006−2009. Additionally, 12.8% HRV cases were co-infected with A/H1N1 2009pdm in 2009−2010, whereas <3% HRV cases were co-infected with H3N2 or H1N1 in 2006−2009 (data not shown).

Other common respiratory viruses, including EV, HPIV, RSV, HMPV, HCoV, and AdV, had no obvious epidemic peaks during or after the dual peaks of A/H1N1 2009pdm and influenza B virus in winter/spring, 2009−2010. This was comparable to the previous years (2006−2009) (Figure 3) [21].

Taken together, these findings suggest that like the seasonal influenza viruses, A/H1N1 2009pdm could also delay the epidemic of the other respiratory viruses, but it could not influence the epidemic of HRV.

### Clinical Features of A/H1N1 2009pdm Infections in 2009/2010

Analysis on demographic and clinical data shows that A/H1N1 2009pdm was more likely to affect young and middle-aged adults, whereas H3N2, H1N1, influenza B virus, and HRV affected people of all age groups tested (Table 2). Statistical analysis of clinical symptoms in 2009/2010 shows that A/H1N1 2009pdm caused mainly cough (90.0%), sore throat (64.0%), and headache (58.6%), whereas seasonal influenza viruses (including H3N2, H1N1, and influenza B virus), mainly caused cough (78.0%), headache (77.5%), sore throat (69.3%), chills (61.0%), and muscle pain (59.2%) (Table 3). The percentage of cough in patients affected by A/H1N1 2009pdm is significantly higher than in patients infected with seasonal infelunza virus and non-influenza respiratory viruses (÷2 = 13.219, P<0.05). Chills were the main symptom in patients infected with seasonal influenza viruses, and were reported significantly less in patients infected with A/H1N1 2009pdm (÷2 = 27.057, *P*<0.05). Non-influenza respiratory viruses frequently induced headache (86.0%), sore throat (66.5%), muscle pain (60.5%), and chills (55.8) (Table 3).

**Table 3:** Demographic and clinical features of A/H1N1 2009pdm, seasonal influenza, and non-influenza respiratory infections in 2009/2010 influenza season.

Parameters	I. A(H1N1) 2009	II. Seasonal influenza viruses	III. Other common respiratory viruses	Statistical analysis		
				I vs II	I vs III	II vs III
No. of patients	261	218	199			
Age range (year)	14−64	14−78	15−83			
No. of age 14−25	127 (48.7) b	75 (34.4)	66 (33.2)	÷2 = 9.898, *P*<0.05	÷2 = 11.129, *P*<0.05	
No. of age 26−65	132 (50.6)	117 (53.7)	117 (58.8)			
No. of age >65	0 (0.0)	24 (11.0)	15 (7.5)	÷2 = 30.250, *P*<0.05	÷2 = 20.337, *P*<0.05	
No. of age unknown	2 (0.8)	2 (0.9)	1 (0.5)			
Age mean/median (year)	27.5/26.0	32.7/27.0	35.5/30.0	*P* = 1.890×10^−5^<0.05(meanvs mean)^c^	*P* = 2.840×10^−9^<0.05(meanvs mean)^c^	*P* = 0.048<0.05(meanvs mean)^c^
Gender (M/F)	123/138(47.1/52.9)	104/114(47.7/52.3)	89/110(44.7/55.3)			
URTIs/LRTIs a	261/0 (100.0/0.0)	218/0 (100.0/0.0)	198/1 (99.5/0.5)			
**Clinical symptoms**						
Cough	235 (90.0)	170 (78)	95 (47.7)	÷2 = 13.219, *P*<0.05	÷2 = 99.648, *P*<0.05	÷2 = 41.078, *P*<0.05
Headache	153 (58.6)	169 (77.5)	160 (80.4)	÷2 = 19.262, *P*<0.05	÷2 = 24.635, *P*<0.05	
Sore throat	167 (64.0)	151 (69.3)	126 (63.3)			
Muscle pain	124 (47.5)	129 (59.2)	111 (55.8)	÷2 = 6.486, *P*<0.05		
Chilly	97 (37.2)	133 (61)	99 (49.7)	÷2 = 27.057, *P*<0.05	÷2 = 7.312, *P*<0.05	÷2 = 5.344, *P*<0.05
Running nose	85 (32.6)	92 (42.2)	82 (41.2)	÷2 = 4.733, *P*<0.05		
Sputum production	88 (33.7)	84 (38.5)	40 (20.1)		÷2 = 10.423, *P*<0.05	÷2 = 16.915, *P*<0.05
Sneezing	52 (19.9)	68 (31.2)	69 (34.7)	÷2 = 8.034, *P*<0.05	÷2 = 12.672, *P*<0.05	
**Peripheral blood tests**						
Mean leukocyte count (×10^9^/L)	6.5±1.7	6.1±1.8	7.2±1.9	*P* = 0.014<0.05^c^	*P* = 1.443×10^−5^<0.05^c^	*P* = 8.592×10^−10^<0.05^c^

a Upper respiratory tract infections/Lower respiratory tract infections;

b Numbers in parentheses are the percentages of infection in total samples;

c Statistical method: student’s *t*-test.

## Discussion

In this study, we characterized the features of A/H1N1 2009pdm epidemic in Beijing and compared the prevalence and clinical characteristics of A/H1N1 2009pdm to seasonal influenza viruses and other respiratory viruses. We found that the influenza epidemic peak (at week 44) caused by A/H1N1 2009pdm pandemic occurred earlier in the winter/spring of 2009−2010 in Beijing than in the winter/spring of 2006−2009 (at week 52 to 2 of next year) (Figure 1, 2 and 3). This finding is in agreement with the observations made by others in China [28]–[30] and to those made in Japan, USA, and European countries, where influenza peaked at week 44, 43, and 46, respectively [6], [11], [31].

Analysis of the annual epidemic pattern revealed a distinctive pattern for the A/H1N1 2009pdm epidemic in Beijing. A prior H3N2 epidemic and a subsequent peak of influenza B were identified with the A/H1N1 2009pdm epidemic (Figure 2 and 3), which is consistent with the results of a previous influenza epidemic in children, in China [32]. However this finding differs from the patterns observed in Shenzhen, China, where epidemics of H3N2 and A/H1N1 2009pdm occurred from May to December 2009 and formed a big epidemic peak [33] and in Hong Kong, China, where the epidemics of H3N2 and A/H1N1 2009pdm occurred simultaneously between May and August 2009 [34]. This epidemic pattern is also different from those observed in USA and European countries [6], [7], [11], [35], where epidemics of H3N2 in summer and influenza B in winter did not occur in 2009. Moreover, compared to nationwide surveillance data from the CNIC, the epidemic peak of influenza in Beijing was higher and appeared 4 weeks earlier than peaks in mainland China (Figure 2). One possible explanation for this observation is that Beijing is a city with dense population in which person-to-person interactions, including interactions with other areas and countries, occur more frequently.

In contrast to observations in Europe [35] and in the USA [7], we found that the predominant viral subtype in the summer of 2009 (week 27−37) in Beijing was H3N2, not A/H1N1 2009pdm (Figure 2 and 3). The reasons for this predominance and its influence on A/H1N1 2009pdm are unclear. It is possible that the summer epidemic of H3N2 hampered the outbreak of A/H1N1 2009pdm. It is also possible that stringent quarantine measures implemented in China to contain A/H1N1 2009pdm in the summer of 2009 delayed the occurrence of A/H1N1 2009pdm and allowed local H3N2 to thrive.

Two influenza peaks, one for A/H1N1 2009pdm and the other for influenza B virus, were observed in Beijing in winter/spring 2009−2010 (Figure 1 and 2). The influenza B virus peak reported by the CNIC did not appear until week 11 with a lower detection rate (27.0%) than we report (68.6%) (Figure 2). This discrepancy may be due to differences in the sampling populations. Our samples were collected from adult patients with suspected respiratory virus infections at the Fever Outpatients Clinic. Pediatric patients were not included. Samples used in the CNIC study were collected from patients with influenza-like symptoms at Internal Departments of sentinel hospitals, including pediatric patients (http://www.cnic.org.cn).

It has previously been suggested that the prevalence of HRV may have delayed the circulation of A/H1N1 2009pdm in France [17]. However, we did not detect such an HRV outbreak prior to the peak of A/H1N1 2009pdm (Figure 3). Meanwhile, in contrast to that observed in previous influenza seasons, A/H1N1 2009pdm did not obviously interfere with the epidemic of HRV and HRV had a relatively long prevalent period (week 40−53) during the epidemic of A/H1N1 2009pdm in the winter/spring, 2009−2010 (Figure 3), indicating that the influence of A/H1N1 2009pdm on the HRV epidemic may differ from that of seasonal influenza viruses. Potential interactions between HRV and influenza viruses could be implied by known biological mechanisms. The receptors of influenza viruses and HRV are sialic acid alpha 2–6 and human intercellular adhesion molecule-1 (ICAM-1), respectively [36], [37]. Viruses do not bind the same receptor in cells, thus, the epidemics of HRV and A/H1N1 2009pdm could exist simultaneously. Our finding that 12.8% HRV cases co-infected with A/H1N1 2009pdm in winter/spring 2009−2010 also suggests this point. The A/H1N1 2009pdm outbreak may have been delayed by the summer epidemic of H3N2 in 2009, in Beijing, because of H3N2 using the same receptors, sialic acid alpha 2–6, as A/H1N1 2009pdm. However, further studies are required to explain the phenomenon that the seasonal influenza viruses H3N2 and H1N1 were not prevalent simultaneously with HRV in 2006−2009.

Seasonal influenza viruses such as H3N2, H1N1, and influenza B virus infected people in all the age groups we tested. However, the A/H1N1 2009pdm predominately infected young and middle-aged adults, consistent with other reports [12], [38]. Our data show that, similar to infection by seasonal influenza viruses and other respiratory viruses, A/H1N1 2009pdm infection is mainly responsible for upper respiratory tract infections. However, we observed different clinical manifestations between infections of A/H1N1 2009pdm and seasonal influenza viruses as well as non-influenza respiratory viruses. We found that A/H1N1 2009pdm infection primarily causes cough, whereas seasonal influenza and other respiratory viruses primarily cause chills and muscle pain, which is consistent with other reports [38], [39].

Our study was limited in several ways. First, all specimens in this study were taken from adult outpatients for whom subclinical infections for respiratory viruses were not assessed. Second, our study did not include paediatric patients. Third, we did not consider any co-infections with bacteria, a relevant factor between infections with different respiratory viruses and symptom severity [40]. Such co-infections could affect the interpretation of common symptoms associated with different infections. In addition, our data were obtained from PCR screening assays and only representative products were verified by DNA sequencing. Overall, however, these limitations do not detract from the main conclusions of this study.

In summary, we observed a distinctive dual peak formed by A/H1N1 2009pdm and influenza B virus in the winter/spring of 2009−2010 with the epidemic of H3N2 in summer in 2009, in Beijing, China. We also found that during the epidemic of A/H1N1 2009pdm, the epidemic of most common respiratory viruses, except HRV, also was delayed. Our findings underscore the complexity of the epidemiological dynamics of A/H1N1 2009pdm and other respiratory viruses and indicate that interference between different viruses might affect their transmissibility between humans [41]. Additional studies are needed to understand the mechanisms and effects of such interference, and these elements should be taken into account for public health decision making to control future influenza epidemics.
